# A Novel QTL in Durum Wheat for Resistance to the Wheat Stem Sawfly Associated with Early Expression of Stem Solidness

**DOI:** 10.1534/g3.119.400240

**Published:** 2019-04-23

**Authors:** Andrea C. Varella, Hongtao Zhang, David K. Weaver, Jason P. Cook, Megan L. Hofland, Peggy Lamb, Shiaoman Chao, John M. Martin, Nancy K. Blake, Luther E. Talbert

**Affiliations:** * Department of Plant Sciences and Plant Pathology, Montana State Univ., Bozeman, Montana 59717; † Department of Land Resources and Environmental Sciences, Montana State Univ., Bozeman, Montana 59717; ‡ Department of Research Centers, Montana State Univ., Bozeman, Montana 59717; § United States Department of Agriculture- Agricultural Research Service, Cereal Crops Research Unit, Fargo North Dakota 58102

**Keywords:** Crop domestication, introgression, insect resistance, genetics, wheat

## Abstract

The wheat stem sawfly (WSS) (*Cephus cinctus* Norton) is a major yield-reducing pest of wheat (*Triticum aestivum* L.). Varieties with pith-filled, or solid, stems provide a measure of resistance by inhibiting larval survival inside the stem. Durum wheat (*Triticum turgidum* L.) has resistance to the wheat stem sawfly even in the absence of known genes for stem solidness. To determine the genetic basis of resistance in durum wheat, a susceptible durum wheat, PI 41353, was identified from among 1,211 landrace accessions from around the world screened in WSS-infested sites. A recombinant inbred line (RIL) population of 105 individuals was developed from a cross of PI 41353 with a typically resistant variety, Pierce. The RIL were screened in a total of three WSS-infested locations in Montana over a two year period. A genetic map was constructed with 2,867 SNP-based markers. A quantitative trait locus (QTL) analysis identified six QTL associated with resistance. An allele from resistant cultivar Pierce at a QTL on chromosome 3A, *Qss.msub-3AL*, caused a 25% reduction in stem cutting. Assessment of near-isogenic lines that varied for alleles at *Qss.msub-3AL* showed that the Pierce allele was also associated with higher stem solidness as measured early in stem development, which is a critical stage for WSS oviposition and larval development. Stem solidness of Pierce and other resistant durum wheat lines largely disappeared later in plant development. Results suggest a genetic mechanism for WSS resistance observed in durum wheat, and provide an additional source of WSS resistance for hexaploid bread wheat.

The recent origin of hexaploid common wheat (*Triticum aestivum* L.) presents a challenge to breeding programs. Hexaploid wheat arose due to hybridization between tetraploid emmer wheat (*T. turgidum* L.) and a wild diploid relative *Aegilops tauschii* as recently as 10,000 years ago (Feuillet *et al.* 2008). Hexaploid wheat, genome designation AABBDD, contains the A and B genomes from tetraploid emmer and the D genome from *A. tauschii*. Ploidy level differences between hexaploid wheat and its progenitors limit the possibility for genetic exchange between species. A manifestation of its recent origin, coupled with limited possibilities for genetic introgression from progenitors, is that genetic diversity within cultivated hexaploid wheat is low (Dubcovsky and Dvorak 2007). The amount of genetic diversity is higher in tetraploid wheat, which includes wild species as well as the primary cultivated emmer wheat and its cultivated conspecific species durum wheat (*T. durum*) (Haudry *et al.* 2007). Thus, breeders have an interest in introgressing useful alleles from tetraploid wheat into hexaploid common wheat.

Barriers to generation of viable progeny from hexaploid by tetraploid wheat crosses include failure to produce F_1_ seed, hybrid necrosis, and unbalanced gametes leading to low fertility (Lanning *et al.*, 2008). Despite these issues, occasional genetic exchange between ploidy levels has occurred during the 10,000 year history of hexaploid wheat (Dvorak *et al.*, 1998; Talbert *et al.* 1998). Additionally, important genes have been introgressed into hexaploid wheat from tetraploid relatives by wheat breeders. For example, the durum wheat cultivar ‘Iumillo’ was used to transfer the stem rust (*Puccinia graminis* f. sp. *tritici*) resistance gene *Sr9g/Yr7* into the widely grown hexaploid cultivar ‘Thatcher’ (Sharma and Gill 1983). An allele for high grain protein concentration was introgressed into hexaploid wheat from tetraploid *T. turgidum*, and has subsequently been incorporated into currently grown varieties (Mesfin *et al.*, 1999; Blake *et al.*, 2014).

Limits to allelic diversity within cultivated hexaploid wheat present challenges for developing cultivars with resistance to insect pests. The wheat stem sawfly (WSS) is a native species that has been an important economic pest of wheat in the Northern Great Plains of North America for more than a century (Beres *et al.*, 2011; Lesieur *et al.* 2016). Another closely related WSS species (*C. pygmaeus* L.) is a historical wheat pest in central Europe (Damania *et al.*, 1997; Korkmaz *et al.*, 2010). Wheat stem sawfly resistant varieties with pith-filled solid stems have been deployed in North America to help control WSS since the 1950s. Most currently grown WSS resistant varieties have a solid-stem trait that can be traced to the landrace accession ‘S-615’ originating from Portugal. A progeny line selected from a cross with S-615, named ‘Rescue’, was the first solid-stem WSS-resistant cultivar released in North America (Platt *et al.*, 1948). A quantitative trait locus (QTL) on chromosome 3B, *Qss.msub-3BL*, has been shown to control most of the variation for stem solidness in crosses between solid and hollow-stem genotypes (Cook *et al.*, 2004). The allele conferring solid stems, *Qss.msub-3BL.b*, originated from Rescue. Based on single nucleotide polymorphisms (SNPs) Cook *et al.* (2017) found the Rescue derived solid-stem haplotype at the *Qss.msub-3BL* locus was common in current WSS-resistant wheat cultivars.

More recent genetic studies have identified a second allele at the *Qss.msub-3BL* locus contributing to stem solidness. This allele was first identified in the cultivar Conan and was designated *Qss.msub-3BL.c* (Sherman *et al.*, 2010; Varella *et al.*, 2017). The *Qss.msub-3BL.c* allele confers a solid-stem phenotype that is different from the phenotype conferred by the Rescue-derived *Qss.msub-3BL.b* allele. Varella *et al.* (2016) developed near-isogenic lines (NILs) that differ for the alleles at the *Qss.msub-3BL* locus. Temporal progression of stem solidness during plant development showed a markedly different pattern of solid-stem expression. Both the Rescue-derived and Conan-derived alleles at *Qss.msub-3BL* locus expressed solid stems early in plant development. The early period of stem elongation coincides with WSS oviposition and development of young larvae in the stem. However, most of the stem solidness was lost later in stem elongation and maturation in NILs with the Conan-derived allele, while stems remained solid throughout stem maturation for NILs with the Rescue-derived allele. Talbert *et al.* (2014) showed that the Conan-derived allele provides a higher level of WSS resistance, leading to reduced oviposition by female WSS, and thus lower stem cutting due to infestation.

The solid-stem trait has been identified in several durum wheat landrace accessions from Turkey, where multiple species of WSS are historic yield-impacting pests (Damania *et al.*, 1997). A QTL for solid-stems has been identified in durum wheat that is orthologous to the *Qss.msub-3BL* QTL in hexaploid wheat (Houshmand *et al.*, 2007). Most durum wheat accessions do not possess the solid-stem *Qss.msub-3BL.b* allele for stem solidness, and have been traditionally classified as hollow-stemmed. However, hollow-stem durum wheat typically has more resistance to WSS than hollow-stem hexaploid wheat (Goosey *et al.*, 2007). Durum wheat appears to possess an allele or alleles for WSS resistance that is not related to the widely used solid-stem trait. Temporal measurements of stem solidness throughout plant development have not been reported for durum wheat.

Genetic dissection of the innate durum wheat resistance to WSS is impeded by the lack of a susceptible line to use as a parent in population development. For this project, we screened a large set of durum wheat landrace accessions to identify a susceptible landrace. The susceptible landrace was crossed to a typically resistant durum wheat cultivar which lacked the solid stem trait conferred by *Qss.msub-3BL* locus. A recombinant inbred line (RIL) population was screened under WSS pressure to identify QTL for resistance. The population was also assessed for temporal progression of stem solidness during plant development. Near-isogenic lines for a QTL conferring resistance were developed to validate its impact on stem solidness in fixed genetic backgrounds. The results have implications for the development of superior WSS-resistant cultivars in both durum and common wheat, and may provide insights into the history of domestication in hexaploid wheat.

## Materials and Methods

### Durum wheat landrace screening

Seeds from 1,221 spring durum wheat landrace accessions were obtained from the USDA-ARS National Small Grains Collection (NSGC). Accessions originated from Africa (n = 126), Asia (n = 604), Europe (n = 488), Oceania (n = 1), and South America (n = 2). Mass screening of landraces was conducted over a period of two years, with each landrace accession being evaluated in a single year. Accessions were planted in late April, at sites naturally infested with WSS located near Loma, MT (48° 04’ 21.96” N, 110° 27’ 41.84” W) in 2013, and Amsterdam, MT (45°45’29.85″N, 111°22’49.32″W) and Loma in 2014. Plots consisted of 10 seeds per entry in unreplicated hills with spacing of 0.6 m between adjacent hills. Check varieties were replicated every 50 hill plots and included a WSS susceptible variety, ‘McNeal’ (PI 574642) or ‘Hank’ (PI 613585), and a solid-stemmed WSS resistant variety, ‘Choteau’ (PI 633974) or ‘Fortuna’ (CItr 13596). Plants were evaluated at maturity for stem solidness using a scoring scale ranging from 1 (completely hollow) to 5 (completely solid), as described by Varella *et al.* (2016). Stems collected from the field were dissected in the laboratory to determine levels of WSS infestation, larval mortality, and parasitism as per Talbert *et al.* (2014). Late heading (Julian heading date >191d) accessions were not collected due to the likelihood that stem elongation occurred after WSS female oviposition. Stem cutting data from the first year of experiments was used to select thirteen durum wheat landrace accessions for further evaluation in replicated trials. Selected accessions were from Ethiopia (n = 1), India (n = 1), Georgia (n = 1), Turkey (n = 8), Peru (n = 1), and Moldova (n = 1). Replicated trials were conducted in Loma and Amsterdam, MT, USA in 2014 and again in Amsterdam in 2015. Hill plots were planted in a randomized complete block design with two replications. Check varieties included the spring wheat line Fortuna and the spring durum wheat ‘Pierce’ (PI 632366). Plots were visually assessed at maturity for percent stem cutting and then stems were collected and dissected for further characterization of WSS parameters as described above.

Results from replicated trials were analyzed using PROC GLM in SAS 9.3 (SAS Institute Inc. 2012) using a model for a randomized block design combined over environments. Entries and locations were considered fixed effects. The LSMEANS statement was used to adjust for unbalanced data from missing plots caused by no germination or insufficient number of stems in the 2015 field trial.

### Recombinant Inbred Line Population

The durum wheat landrace PI 41353 was found to have a high level of infestation and stem cutting. A total of 105 RILs were developed from the cross Pierce/PI 41353. Pierce showed typical durum wheat resistance to the WSS. The RIL were derived by single seed descent from F_2_ to F_5_ generations, at which point a single plant was used as seed source for each line. Seed was advanced in bulk for each line in subsequent generations. The populations were planted in three WSS-infested sites, including Amsterdam, MT (45°45’30” N, 111°22’49” W) in 2016 and 2017, and Big Sandy, MT (48° 15’ 46” N, 110° 22’ 11” W) in 2017. Hill plots were established as ten seeds per plot spaced 0.6 m apart. Trials were planted as randomized complete block designs with three replications. Check varieties included solid-stemmed hexaploid wheat Choteau and hollow-stemmed hexaploid wheat ‘Reeder’ (PI 613586), along with parents PI 41353 and Pierce.

A visual estimate of percent stem cutting was recorded for each hill plot at the end of the season. Additionally, every stem in each plot was collected for dissection to determine the presence and eventual fate of WSS larvae. Stem dissection revealed varying levels of parasitism by endemic natural enemies (Runyon *et al.* 2002; Sherman *et al.* 2010), which was recorded for each plot. Key parameters derived from stem dissection included the percentage of stems containing larvae or eggs (infestation), the percentage of dead larvae minus the percentage of those killed by parasitoids (mortality), and the percentage of stubs, or cut stems. Plant heading date were obtained for the two Amsterdam environments. Data for each response variable for this multi-location trial was analyzed via analysis of variance using a model for a randomized block design combined over locations with PROC GLM in SAS 9.3 (SAS Institute Inc. 2012). Environments and entries were considered fixed effects. The LSMEANs statement was used to calculate entry means due to the occurrence of missing plots. For one location (Amsterdam 2016), entries were also scored for the number of internodes with boring injury for all infested stems. This data were analyzed by PROC GLM with entries as fixed effects.

The RILs were also planted as unreplicated 3-m rows at a non-WSS infested site in Bozeman MT (45° 40’ 33.6’’ N, 111° 9’ 25.2’’ W) over a two-year period. This trial allowed assessment of both early and late stem solidness and plant height as described by Varella *et al.* (2016). Early stem solidness was assessed by collecting the main stems of three plants of each plot approximately 35 days after planting when plants were at Zadoks 37 (at least two measurable internodes) (Zadoks *et al.* 1974). Internodes were scored on a 1 to 5 scale, with 1 being hollow and 5 being solid. Final stem solidness of each main stem was calculated by total solidness score divided by total number of internodes. Late stem solidness was assessed by collecting the main stems of three plants of each plot at maturity (near Zadoks 77) in late August. Stems were dissected and each internode was rated for stem solidness as described above. Analysis of variance was conducted using each year as a replicate to test for significance of entry effects on stem solidness. Entries were considered fixed effects. Correlation analysis between stem solidness and the mean for WSS resistance traits measured in three WSS-infested locations was performed with PROC CORR in SAS 9.3 (SAS Institute Inc. 2012).

### 90K iSelect genotyping

The RIL population was genotyped using the Illumina 90K iSelect assay (Wang *et al.*, 2014). Data analysis was conducted using Illumina’s GenomeStudio 2011 v1 software (Illumina, Inc., San Diego CA, USA). Allele call for each SNP was done manually. Markers with more than 10% missing genotypes, monomorphic or showing significant distortion at the 0.05 level after Bonferrroni correction were discarded.

### Sequence-based SNP genotyping

Genomic DNA from RIL populations derived from Pierce/PI 41353 were quantified using PicoGreen (LifeTechnologies) and normalized to ∼50 ng uL-1 of DNA per line. Libraries for sequencing (95-plex) were prepared according to Saintenac *et al.* (2013) using the *Pst*I/*Mse*I combination of enzymes. Sequencing was performed on an Illumina HiSeq2500 platform with 100-bp paired-end sequence reads. The analysis pipeline was conducted using TASSEL software version 4.0 (Glaubitz *et al.* 2014). Briefly, tag counts were generated and merged using default parameters with the FastqToTagCountPlugin and MergeMultipleTagCountPlugin, respectively. Bowtie 2 version 2.2.9 (http://bowtie-bio.sourceforge.net/bowtie2/index.shtml) was used to align tags to the wheat pseudo-reference genome (accessible at: ftp://ftp.ensemblgenomes.org/pub/plants/release-31/fasta/triticum_aestivum/dna/) (IWGSC, 2014). The output of the alignment was converted to a “Tags On Physical Map” (TOPM) file by the SAMConverterPlugin. The SeqToTBTHDF5Plugin and ModifyTBTHDF5Plugin were used to generate a “Tags by Taxa” (TBT) file containing sorted and demultiplexed reads. SNPs were called using the DiscoverySNPCallerPlugin with the following non-default parameters: Minimum value of F (inbreeding coefficient = 1-Ho/He, where Ho is the observed heterozygosity and He is the expected heterozygosity) [mnF]: 0.8, Minimum minor allele frequency (default: 0.01) [mnMAF]: 0.02, and Minimum minor allele count (default: 10) [mnMAC]: 100,000. Duplicate sites were merged with the MergeDuplicateSNPsPlugin. Finally, SNPs with low taxon coverage and low minor allele frequency were filtered out with the GBSHapMapFiltersPlugin and the non-default parameters: Minimum site coverage (default: no filter) [mnScov]: 0.2, Minimum minor allele frequency (default: 0.0) [mnMAF]: 0.01, and Maximum minor allele frequency (default: 1) [mxMAF]: 0.5.

### Genetic linkage map construction and QTL analysis

Linkage map construction for the Pierce/PI 41353 RIL population was conducted using R/qtl (Broman *et al.*, 2003) and R/ASMap (Taylor and Butler 2014) packages in R (Broman and Sen 2009). Polymorphic markers with more than 25% missing data or significant Mendelian segregation distortion (*χ2* test, *P* < 1.0e^-7^, d.f. = 1) were excluded. Co-segregating markers were also discarded. The *mstmap* function (Wu *et al.* 2008) from R/ASMap package was used to group and order markers. Map distances (cM) were calculated using the Kosambi function with a significance threshold of *p.value = 1e^-7^* for linkage group formation. A heat map of estimated recombinant fractions and LOD scores was used for checking marker order on each linkage group. Standard interval mapping (Broman *et al.*, 2003) was conducted using the *scanone* function and the Haley-Knott regression method. Significance thresholds (*P* < 0.05) for LOD scores were determined using permutations with 1,000 replications. Phenotypic and genotypic data used for the QTL analysis is in Supplementary Table S1.

### Development of Near-Isogenic Lines for a 3A QTL and stem solidness progression experiment

The heterogeneous inbred family method (Haley *et al.*, 1994; Pumphrey *et al.*, 2007) was used to develop NIL for a QTL identified on chromosome 3A for early solidness and WSS cutting. Three Wheat iSelect 90K chip (Wang *et al.* 2014) SNP markers associated with the 3A QTL were converted to kompetitive allele specific PCR (KASP) markers (LGC Biosearch Technologies, Middlesex, UK) . KASP marker sequences were obtained from the Wheat iSelect 90K designed markers available from PolyMarker (http://polymarker.tgac.ac.uk), The Genome Analysis Center of John Innes Center). The KASP genotyping system was used for KASP assays, following the LGC protocol (https://www.biosearchtech.com). Reactions were performed on the Bio-Rad CFX96 touch real-time PCR detection system (Bio-Rad, Hercules, CA), and allelic calls were made using the Bio-Rad CFX manager software version 3.1 (Bio-Rad, Hercules, CA). The KASP markers were used to screen F_5_ plants from each RIL of Pierce/ PI 41353 population. Four F_5_ plants heterozygous for the 3A QTL were identified. The four heterozygous individuals were allowed to self-pollinate to generate F_6_ progeny. The KASP markers were used to identify homozygous F_6_ individuals. These homozygous F_5_-derived F_6_ plants were the source of paired NIL containing either the Pierce or PI 41353 allele at the chromosome 3A QTL. Each NIL pair is designated as a family. Family CPSD9-6 included three lines with the PI 41353 allele and three lines with the Pierce allele. Families CPSD9-22, CPSD9-88 and CPSD9-101 each had one line of each allele type. A total of six NIL pairs was used in a time-course experiment on the progression of stem solidness in a greenhouse study in the Plant Growth Center at Montana State University (MSU-PGC, Bozeman, MT). The trial also included parents PI 41353 and Pierce, as well as hexaploid wheat checks Conan, Choteau and Reeder. Three additional durum wheat lines included resistant landraces PI 91956 and PI 178678, and resistant durum wheat variety Mountrail (PI 607540). The NIL and check varieties were planted at four different dates in the fall of 2018, with three pots of each genotype per planting date The average value of three plants per pot was used for analysis. The experiment was designed as randomized complete block, with each planting date serving as a replication. Plants were grown under a 16-hour day photoperiod with the day/night temperature of 22°/20°, watered regularly and fertilized twice a week beginning at the 3 leaf stage with 100ppm Peter’s Professional 20-20-20 Fertilizer.

The progression of stem solidness was assessed by dissecting internodes of the main stem at three growth stages, including Zadoks stage 37 (flag leaf just visible; about 42 days after planting), Zadoks stage 49 (first awns visible; approximately 48 days after planting) and Zadoks stage 77 (late milk; approximately 65 days after planting). One of the three pots of each genotype per replication was used for stem dissection for each of the three stages. Stem solidness was scored using a scale ranging from 1 (completely hollow) to 5 (completely solid), as described by Varella *et al.* (2016). Final stem solidness of each main stem was calculated by total solidness score divided by total number of internodes. PROC GLM in SAS 9.3 (SAS Institute Inc. 2012) was used to conduct an analysis of variance to determine allele effects within each NIL family and combined over families, where allele type was considered a fixed effect. To assess whether stem solidness scores declined over time, the Slice command in PROC GLM (SAS Institute Inc. 2012) was used to determine differences between solid stem scores at Zadoks 37 and Zadoks 77 for each entry. Entries and Zadoks stage were considered fixed effects.

### Data Availability

Supplementary material is available at GSA figshare portal. Table S1 contains phenotypic and genotypic data used for the QTL analysis. Table S2 contains sequences for three KASP markers used to develop NIL for a WSS-resistance QTL. Figure S1 is a graphic genetic map with QTL locations. Supplemental material available at FigShare: https://doi.org/10.25387/g3.7934615.

## Results

Of the 1,221 durum wheat landraces planted in 2013 and 2014, 571 accessions were collected and dissected over the course of two years and three trials from WSS-infested sites in Montana. Approximately 10% of the plots were missing among the three trials. Additionally, late heading plots were not collected and in 2013 only uncut plots were collected. Very little cutting was observed in most of the durum wheat landraces. Stem infestation in the durum wheat was consistently low, with mean values of 2.8% in 2013, 5.5% in 2014, and 12.8% in 2015 (data not shown). Nevertheless, infestation ranged from 0 to 44%, and stem cutting ranged from 0 to 29.7%, with a mean value of 0.73%.

Based on the initial screening nurseries, thirteen durum wheat accessions that varied for stem cutting in the screening trials were entered into replicated trials grown over three environments (Table 1). Most accessions had less than 10% infested stems (Table 1). The level of cutting in most of the durum wheat landrace accessions was not different from that observed for the hexaploid wheat check cultivar Fortuna. Fortuna is a historically important solid-stemmed cultivar grown due to its resistance to the WSS (Lebsock *et al.*, 1967). Fortuna is also a standard height cultivar similar to the landrace durum wheat accessions. A single accession from India, PI 41353, had a significantly (*P* < 0.05) higher level of infestation (31.7%) than the other durum wheat landraces and the resistant common wheat Fortuna (Table 1). Pierce had low stem cutting due to the WSS similar to other durum wheat lines, though initial infestation was higher (Table 1). Pierce had only 1% cutting *vs.* 13% for PI 41353.

**Table 1:** Analysis of variance and mean values for wheat stem sawfly traits in selected durum wheat landrace accessions averaged over three Montana environments in 2016 and 2017

Origin	PI Number	Infestation (%)	Mortality*^a^* (%)	Stem cutting*^b^* (%)
India	PI 41353	31.7	37.8	13.3
Georgia	PI 61111	5.2	73.8	0.3
Moldova	PI 61185	5.8	50	0.4
Peru	PI 91956	3.6	30.6	0.9
Ethiopia	Cltr 14434	0.0	.	.
Turkey	PI 166524	8.0	57.4	1.1
Turkey	PI 166955	2.3	50	0.4
Turkey	PI 167436	10.9	45	3.5
Turkey	PI 173487	3.5	62.5	0
Turkey	PI 177947	5.4	74.2	0.9
Turkey	PI 178048	4.0	55.6	0
Turkey	PI 341735	2.7	100	0
Turkey	PI 178678	13.5	100	0
USA (durum check)	PI 632366	12.3	59.6	1.0
USA (wheat check)	PI 13596	13.4	41.2	0.32
P value – Among accessions		0.0005	0.10	<0.0001
P value- accession x environment		0.76	0.27	0.27
LSD (0.05)		10.9	54.7	3.6

a Mortality is calculated as total larval mortality in the stem minus the number of larvae killed by parasitoids divided by the total number of infested stems.

b Stem cutting was determined by counting stubs at the time of stem dissection.

A total of 105 RILs were developed by single seed descent from a cross between resistant Pierce and susceptible PI 41353. Assessment of stem solidness over two years in a non-WSS infested site showed that the RILs differed significantly for stem solidness measured early in plant development (Zadoks 37) with mean solidness score of 3.5, where 1 is completely hollow and 5 is completely solid (Table 2). The parental line Pierce had a solid stem score of 3.7 *vs.* 3.1 for PI 41353 (*P* = 0.07) in these trials. Solid-stem measurements at plant maturity (Zadoks 77) also varied significantly among the progeny, though the amount of variation was lower than that for the solid stem measurements early in plant development. Pierce had a solid stem score at maturity of 2.6 *vs.* 2.5 for PI 41353 (*P* > 0.05). The RILs also varied significantly for all WSS resistance traits, including percent infestation, percent mortality, and field and laboratory measurements of stem cutting.

**Table 2:** Analysis of variance and recombinant inbred line population mean and range for WSS-related traits for a set of 105 RIL developed from a Pierce/PI 41353 cross

	Temporal Solid-Stem Measurements		Stem Cutting (%) Field Assessment*^b^*			Stem Cutting (%) Lab Assessment*^d^*
	Zadoks 32 (1-5)*^a^*	Zadoks 77 (1-5)*^a^*		Infestation (%)	Mortality*^c^* (%)	
Number of Environments	2	2	3	3	3	3
RIL Mean	3.5	2.7	19.2	39.7	47.2	19.6
RIL Range	2.8-4.6	2.1-3.6	6.9-34.4	22.7-56.7	17.1-66.6	7.5-31.5
P value – Among RIL	<0.0001	<0.0001	<0.0001	<0.0001	<0.0001	<0.0001
P Value – RIL X Environment	ND	ND	0.18	0.07	<0.0001	0.002

a Scale of 1-5, where 1 is hollow and 5 is completely solid.

b Visual assessment of percent cut stems in the field.

c Mortality is calculated as total larval mortality in the stem minus the number of large larvae killed by parasitoids divided by the total number of infested stems.

d Laboratory assessment on collected plots at the time of dissection.

A correlation analysis of stem solidness data and WSS resistance was conducted based on the means over sites (Table 3). Solid-stem scores early in plant development showed a significant negative correlation with percent WSS infestation and both measures of stem cutting. Early measurement of stem solidness (Zadoks 37) was positively correlated with percent mortality (*P* < 0.05). However, solid-stem scores at plant maturity (Zadoks 77) were not significantly correlated with any measurements of WSS resistance (Table 3).

**Table 3:** Correlation coefficients and level of significance between stem solidness and WSS resistance traits in a set of 105 recombinant inbred lines from a Pierce/PI 41353 cross. The P value is given parenthetically

Temporal Solid-Stem Measurements	Infestation	Mortality	Stem Cutting – Field Assessment*^a^*	Stem Cutting – Lab Assessment*^b^*
Zadoks 37	−0.33 (0.0008)	0.19 (0.05)	−0.38 (<0.0001)	−0.41 (<0.0001)
Zadok 77	0.08 (0.39)	0.07 (0.43)	-0.10 (0.32)	−0.02 (0.83)

a Visual assessment of percent cut stems in the field.

b Laboratory assessment on collected plots at the time of dissection.

A genetic map was constructed for the population of 105 Pierce/PI 41353 RILs. The sequence-based SNP genotyping procedure yielded 4,375 polymorphic markers, from which 974 were mapped along with 1,893 SNP markers derived from the Illumina 90K iSelect array. Average spacing was 1.8 cM with a marker density of 1.7 cM/marker. Linkage groups varied in number of markers and marker density, with linkage group 4B showing the smallest number of markers (n = 93). The genetic map is shown in Supplementary Table S1.

A QTL analysis was conducted based on data combined over locations. The solid stem data were collected from two non-WSS infested locations, while the WSS resistance data were collected from three WSS infested nurseries. Alleles for WSS resistance were contributed by both parents. Least square means over three WSS infested locations (Table 4) showed a significant QTL on chromosome 3A, designated *Qss.msub-3AL*, with Pierce contributing the *Qss.msub-3AL.b* allele for low stem cutting based on field measurement. Landrace PI 41353 contributed the *Qss.msub-3AL.a* allele for greater stem cutting. The number of internodes bored by the WSS for each infested stem, assessed only in the Amsterdam trial in 2016, showed a difference (*P* < 0.05) between RIL with alternative alleles at *Qss.msub-3AL*. The RIL with the Pierce allele had an average of 2.0 internodes bored per infested stem, while the number of bored internodes was 2.3 for RIL with the PI 41353 allele (data not shown).

**Table 4:** QTL identified in the Pierce/PI 41353 recombinant inbred line population of 105 individuals based on means over three sites for WSS resistance traits and two sites for stem solidness measurements

Trait	Peak Marker	QTL Number*^a^*	Chromosome	Position (cM)	Confidence Interval (cM)*^d^*		LOD	Allele mean		Coincident QTL
					Low	High		Pierce	PI 41353	
Infestation (%)	RAC875_c9386_358	1	1A	12.7	0	28	3.99	37.7	42.6	
Infestation (%)	tplb0044i19_1180	2	7B	98.2	80	101	3.77	42.2	37.4	
Mortality (%)	S8_130643035	3	3B	222	215	312	3.52	44.4	51.9	
Early Stem Solidness (Zadoks 37)	Ku__c19285_555	4	3A*^g^*	144	139	230	3.77	3.7	3.3	Height*^e^*
Stem Cutting – Field Assessment (%)*^b^*	RAC875_c19860_373	4	3A*^g^*	150.9	143	224	4.01	16.6	21.6	Height*^e^*
Stem Cutting –Lab Assessment (%)*^c^*	wsnp_Ra_c407_862316	5	2B	8	0	16	3.72	17.3	21.1	Heading Date*^f^*
Late Stem Solidness (Zadoks 77)	GENE-1351_291	6	6B	274	209	282	5.1	2.8	2.6	

a QTL numbers as depicted on graphical genetic map (Supplemental Figure 1)

b Visual assessment of percent cut stems in the field.

c Laboratory assessment on collected plots at the time of dissection.

d Confidence intervals were calculated by the Bayes credible interval method (Broman and Sen 2009).

e The Pierce allele was associated with reduced height of 6.2 cm.

f The Pierce allele was associated with delayed heading of 2.5 days.

g This QTL is designated *Qss.msub-3AL*.

A QTL was also detected at *Qss.msub-3AL* for solid-stems measured early in plant development based on data averaged across two non-WSS infested sites (Table 4). The Pierce *Qss.msub-3AL.b* allele was associated with increased stem solidness. The Pierce allele on chromosome 3A was also associated with decreased plant height (data not shown). An allele for lower WSS cutting (lab assessment) on chromosome 2B was contributed by Pierce. This allele was associated with a significant delay in heading (data not shown). Pierce also contributed an allele for low WSS infestation for a QTL on chromosome 1A. The susceptible parent PI 41353 contributed alleles for low WSS infestation (chromosome 7B) and WSS high mortality (chromosome 3B) (Table 4). A graphic genetic map with QTL locations is shown in Supplementary Figure S1.

The association of the Pierce *Qss.msub-3AL.b* allele with reduced WSS stem cutting and increased stem solidness early in plant development prompted the development of NIL that varied for alleles at the 3A QTL. Four heterozygous individuals were identified in the F_5_ generation of the Pierce/ PI 41353 RIL population. These were used to derive the homozygous classes of NIL based on the ‘heterogeneous inbred population’ method (Haley *et al.*, 1994; Pumphrey *et al.*, 2007) using KASP markers shown in Supplemental Table S2. Paired NILs were tested for temporal solid-stem variation across plant developmental stages in the greenhouse (Table 5). The Pierce *Qss.msub-3AL.b* allele was associated with higher solid stem scores early in plant development (Zadoks 37) across all four NIL comparisons, with a mean solid stem score of 2.9 *vs.* 1.9 for the PI 41353 allele. Increased stem solidness was also associated with the Pierce *Qss.msub-3AL.b* allele as the stem matured (Zadoks 49 and Zadok 77), though the difference between the Pierce allele and the PI 41353 allele decreased. Measurements taken at Zadoks 77 (soft dough stage) showed that the Pierce allele resulted in a solid-stem score of 1.9 *vs.* 1.5 for the PI 41353 allele.

**Table 5:** Analysis of variance of the progression of stem solidness among paired NIL that varied for alleles at *Qss.msub-3AL*

Family	Allele	Temporal Solid-Stem Measurements*^a^*		
		Zadoks 37	Zadoks 49	Zadoks 77
CPSD9-6	Pierce	3.62	2.73	2.12
	PI 41353	2.61	2.18	1.58
NIL P Value		<0.0001	<0.0001	<0.0001
CPSD9-22	Pierce	2.90	2.37	1.84
	PI 41353	1.61	1.69	1.52
NIL P Value		<0.0001	0.0007	0.0111
CPSD9-88	Pierce	2.15	2.22	1.93
	PI 41353	1.72	1.64	1.57
NIL P Value		0.11	0.0034	0.0044
CPSD9-101	Pierce	2.81	2.37	1.94
	PI 41353	1.71	1.79	1.42
NIL P Value		0.0002	0.0034	<0.0001
Mean across families	Pierce	2.9	2.4	1.9
	PI 41353	1.9	1.8	1.4
NIL P value		<0.0001	<0.0001	<0.0001

a Scale is 1 to 5, with 1 being hollow and 5 being completely solid.

Temporal measurements of stem solidness were also assessed in other tetraploid and hexaploid lines (Table 6). The hexaploid wheat Choteau carries the Rescue-derived solid-stem *Qss.msub-3BL.b* allele while hexaploid Conan has the alternative solid-stem *Qss.msub-3BL.c* allele. Both showed significantly greater stem solidness at all stages in comparison to the hexaploid hollow stem variety Reeder which has the hollow stem *Qss.msub-3BL.a* allele. Analysis of variance comparing scores at Zadoks 37 and Zadoks 77 showed that stem solidness declined over time in both Choteau and Conan (*P* < 0.05) while Reeder maintained hollow stems throughout the time course (*P* > 0.05). Among the four tetraploid wheats tested, PI 41353 showed the lowest level of stem solidness at Zadoks 37, significantly lower than Pierce, PI 91956, and PI 178678. Stem solidness for PI 41353 at Zadoks 49 was lower than that for all four of the other durum wheat accessions. Stem solidness at Zadoks 77 was lower in PI 41353 than for three of the four other durum wheat accessions.

**Table 6:** Means of stem solidness progression for hexaploid and tetraploid checks

ID	Ploidy	Temporal Solid-Stem Measurements*^a^*		
		Zadok 37	Zadok 49	Zadok 77
Choteau	Hexaploid	3.24	2.40	2.47
Conan	Hexaploid	3.71	2.50	2.13
Reeder	Hexaploid	1.56	1.58	1.55
Pierce	Tetraploid	3.00	2.58	1.97
PI 41353	Tetraploid	1.93	1.64	1.39
Mountrail	Tetraploid	2.35	2.10	1.44
PI 91956	Tetraploid	3.92	2.64	2.99
PI 178678	Tetraploid	2.99	2.61	2.17
P value *^b^*		<0.0001	<0.0001	<0.0001
LSD		0.54	0.43	0.26

a Scale is 1 to 5, with 1 being hollow and 5 being completely solid.

b P value is from analysis of variance of means including both NIL and checks (n = 21).

## Discussion

Crossing barriers due to ploidy differences have limited the amount of genetic diversity in hexaploid wheat relative to its ancestors. Diversity present in progenitors can be accessed through directed crossing programs, either through development of synthetic hexaploids from tetraploid wheat and diploid *Ae. tauschii*, or through direct crossing of tetraploid wheat with hexaploid wheat. Lanning *et al.* (2008) showed that most crosses between durum wheat and hexaploid wheat produce low numbers of viable progeny, though some crosses have a higher success rate. Kalous *et al.* (2015) used this information to develop both tetraploid and hexaploid RIL populations from tetraploid by hexaploid crosses. In their study, favorable alleles for yield related traits could be identified from the durum wheat parent in a hexaploid background. However, the overall performance of the hexaploid RIL was inferior to both the durum or hexaploid wheat parent. Thus, introgression of specific alleles using marker-assisted selection from durum wheat into hexaploid wheat may be necessary for successful utilization of durum wheat germplasm to improve hexaploid wheat.

An example of a potentially useful trait from durum wheat is resistance to WSS. Resistance to WSS in hexaploid wheat has historically depended upon alleles for solid stems at the *Qss.msub-3BL* locus originally introduced into the cultivar Rescue from a Portuguese landrace, S-615 (Platt *et al.*, 1948). An orthologous locus controlling solid stems in durum wheat has been designated *Sst1* (Houshmand *et al.*, 2007). However, even in the absence of stem solidness due to *Sst1*, durum wheat has resistance to WSS relative to hollow stem hexaploid wheat (Goosey *et al.*, 2007). The genetic basis for this resistance has not been determined. An impediment to identifying the genetic source of WSS resistance in durum has been the lack of a susceptible durum wheat to use as a parent in crosses to resistant durum wheat. The screening of a large number of durum wheat landraces for this research allowed identification of a susceptible landrace, PI 41353.

The RIL population developed from a cross between WSS resistant Pierce and WSS susceptible PI 41353 allowed identification of an allele from Pierce at *Qss.msub-3AL* that had high stem solidness early in plant development and was associated with reduced stem cutting by WSS. The degree of solid stems early in plant development was positively correlated to all measures of WSS resistance, causing decreased infestation, increased larval mortality, and decreased stem cutting. Varella *et al.* (2017) showed high levels of stem solidness early in plant development attributable to the *Qss.msub-3BL.c* Conan-derived allele also resulted in lower infestation. There was no correlation between stem-solidness measured at plant maturity and WSS resistance in the present study. Development of NILs using KASP markers linked to *Qss.msub-3AL* allowed verification of the solid-stem phenotype caused by the allele from Pierce. The Pierce *Qss.msub-3AL.b* allele was associated with solid stems early in plant development in the NIL, but the solid-stem level declined as the plant progresses to maturity. The early expression of stem solidness appears to be critical for conferring resistance to the WSS.

The durum wheat solid stem *Qss.msub-3AL.b* allele may be useful to enhance resistance in hexaploid wheat. The first step in this process is to move the durum wheat allele into a hexaploid wheat background. This step has likely already been accomplished through previous durum by hexaploid crossing programs. Table 6 indicates that the five durum wheat lines assessed in this study have solid stems early in plant development but this declines as the plant matures. Kalous *et al.* (2015) developed 177 hexaploid wheat lines from hexaploid by durum wheat crosses. A total of 66 of these lines contained the allele from durum at *Qss.msub-3AL.b*. Development of RIL populations using hexaploid lines with the durum wheat allele at *Qss.msub-3AL.b* will allow determination of the durum wheat solid stem phenotype and its effect on WSS resistance in a hexaploid wheat background.

The stem solidness and WSS resistance phenotype expressed by the durum wheat *Qss.msub-3AL* QTL is similar to the hexaploid wheat Conan-derived *Qss.msub-3BL.c* allele (Table 6; Varella *et al.*, 2016). The Conan-derived *Qss.msub-3BL.c* allele results in high stem solidness early in plant development and a high level of resistance to WSS (Talbert *et al.*, 2014). The solid stem phenotype observed early in plant development has not been used as a target for breeding for resistance to the WSS. This is largely because screening for solid-stems has typically occurred near plant maturity (Wallace *et al.*, 1969), and the phenotype of solid-stems early in plant development that then decreases as the plant matures would be missed. Screening for solid stems late in plant development is efficient providing the solid stem is due to the Rescue derived *Qss.msub-3BL.b* allele. This allele provides stable stem solidness throughout the life of the plant. However, the durum wheat *Qss.msub-3AL*.*b* allele and the Conan-derived *Qss.msub-3BL.c* allele do not provide an easily detectable increase in stem solidness late in plant development. Thus, a revised strategy for detecting the phenotype or the presence of the favorable allele is needed.

One method for detecting early stem solidness among a large number of breeding lines is to assay plants by a longitudinal sectioning of the elongating main stem. This is problematic in large scale applied breeding for several reasons. First, the tissue is fragile and requires more care than may be possible in nurseries containing thousands of segregating progeny rows. Second, sampling is destructive to the plant. Finally, stem elongation does not occur at the same time for all progeny, leading to the need to revisit nurseries several times. Conducting stem dissection by cross-sectioning at maturity with scissors avoids all of these problems, but does not allow detection of early stem solidness. It is likely that it is most efficient to use DNA-based markers to detect alleles for early stem solidness. Molecular markers linked to stem solidness can be used in breeding programs to facilitate marker assisted selection and gene pyramiding, which allows screening of large population even in the seedling stage with just a piece of leaf tissue. Among different kinds of molecular markers, KASP, owing its low cost, high throughput, and specificity, has been extensively used in SNP genotyping. Our study revealed three KASP markers that can be used to detect *Qss.msub-3AL* in the development of varieties resistant to WSS.

In conclusion, a cross was made between a typically WSS-resistant durum wheat Pierce and a rare susceptible durum wheat landrace PI 41353. Analysis of RIL revealed several QTL for WSS resistance, including *Qss.msub-3AL.b* which was also associated with early stem solidness. Early stem solidness was significantly correlated with low WSS infestation, high WSS mortality and low levels of stem cutting. Development of NIL for *Qss.msub-3AL.b* showed the Pierce allele caused a high level of early stem solidness, which declined during stem development. These results provide an explanation for the inherent resistance to WSS observed in most durum wheat accessions. Additionally, the KASP markers developed for the durum wheat allele for early stem solidness will be useful for developing hexaploid wheat cultivars with resistance to the wheat stem sawfly.
